# Overexpressed HDAC8 in cervical cancer cells shows functional redundancy of tubulin deacetylation with HDAC6

**DOI:** 10.1186/s12964-018-0231-4

**Published:** 2018-05-02

**Authors:** G. R. Vanaja, Hemalatha Golaconda Ramulu, Arunasree M. Kalle

**Affiliations:** 1 Department of Animal Biology, School of Life Sciences, University of Hyderabad, Hyderabad, TS 500046 India; 2 Rue du RIA, 13003 Marseille, France

**Keywords:** HDAC8, Alpha tubulin, Tubulin deacetylase, HDAC6, Cervical cancer

## Abstract

**Background:**

Histone deacetylases (HDACs) are involved in epigenetic gene regulation via deacetylation of acetylated lysine residues of both histone and non-histone proteins. Among the 18 HDACs identified in humans, HDAC8, a class I HDAC, is best understood structurally and enzymatically. However, its precise subcellular location, function in normal cellular physiology, its protein partners and substrates still remain elusive.

**Methods:**

The subcellular localization of HDAC8 was studied using immunofluorescence and confocal imaging. The binding parterns were identified employing immunoprecipitation (IP) followed by MALDI-TOF analysis and confirmed using *in-silico* protein-protein interaction studies, HPLC-based in vitro deacetylation assay, intrinsic fluorescence spectrophotometric analysis, Circular dichroism (CD) and Surface Plasmon Resonance (SPR). Functional characterization of the binding was carried out using immunoblot and knockdown by siRNA. Using one way ANOVA statistical significance (*n* = 3) was determined.

**Results:**

Here, we show that HDAC8 and its phosphorylated form (pHDAC8) localized predominantly in the cytoplasm in cancerous, HeLa, and non-cancerous (normal), HEK293T, cells, although nucleolar localization was observed in HeLa cells. The study identified Alpha tubulin as a novel interacting partner of HDAC8. Further, the results indicated binding and deacetylation of tubulin at ac-lys40 by HDAC8. Knockdown of HDAC8 by siRNA, inhibition of HDAC8 and/or HDAC6 by PCI-34051 and tubastatin respectively, cell-migration, cell morphology and cell cycle analysis clearly explained HDAC8 as tubulin deacetylase in HeLa cells and HDAC6 in HEK 293 T cells.

**Conclusions:**

HDAC8 shows functional redundancy with HDAC6 when overexpressed in cervical cancer cells, HeLa, and deacetylaes ac-lys40 of alpha tubulin leading to cervical cancer proliferation and progression.

## Background

Histone deacetylases (HDACs) are class of enzymes known to deacetylate the ε-amino groups of acetylated lysine residues of both histone and non-histone proteins [1] and are divided into four different classes: Class I: HDACs 1, 2, 3, and 8, Class II: HDACs 4,5, 6, 7, 9, 10, Class III: Sirtuins, SIRT1-SIRT7, and Class IV: HDAC11 [2]. Zinc dependent hydrolases class of HDACs (I, II and IV) are commonly referred to as “Classical” HDACs [3]. Given the role of HDACs in epigenetic gene regulation and protein activity regulation, identification and characterization of endogenous substrates of HDAC enzymes is a promising area of HDAC research [1].

HDAC8, the best structurally characterized [4] of all HDACs is known to be the first human HDAC to be crystallized with bound inhibitors [5–8]. Histone proteins such as H2A/H2B, H3, and H4 and non-histone proteins such as p53, α-actin, CREB etc. have been identified as substrates of HDAC8 [9]. Overexpression of HDAC8 is clinically observed in some of the adult tissue cancers like colon, breast, lung, pancreas, liver and childhood neuroblastoma [10, 11]. Knockdown of HDAC8 using siRNA has shown to inhibit human lung, colon, and cervical cancer proliferation [6]. Interestingly, HDAC8 knockdown did not affect the overall histone acetylation [11]. In view of the role of identified HDAC8 substrates in cellular homeostasis and the role of HDAC8 in tumorigenesis, it is important to identify and characterize the functional significance of other novel protein substrates of HDAC8.

In the present study an attempt is made to identify and characterize novel HDAC8 substrates.

## Methods

### Materials

Forskolin was purchased from Sigma (USA). PCI-34051 and Tubastatin were procured from Cayman chemicals. DMEM, FBS, 1% Penicillin & Streptomycin Antibiotics were obtained from Himedia. Monoclonal antibody of HDAC8 (A-4008) was obtained from Epigenetik, Beta actin ab8227 (Abcam), GAPDH (MA5–15738), Alpha tubulin (B-5-1-2) and anti-acetylated alpha tubulin (Cat no: 322700) were purchased from Thermo scientific Life technologies respectively. Protein A Agarose beads were obtained from Santa Cruz. Poly-L-Lysine (Sigma P8920), 4′, 6-Diamindino-2-phenylindole dichloride DAPI as a nuclear stain (Sigma), Alexa Fluor® 555 Dye (Thermo Fischer Scientific). HDAC8 FLUOR DE LYS fluorometric assay kit (BML-AK518–0001) was purchased from Enzo life sciences. Propidium iodide was purchased from Himedia. GST Column (GE Healthcare 17–5132-01), Custom synthesized peptides of alpha tubulin 33–46 amino acids, Acetylated alpha tubulin peptide, at Lys40 DGQMPSDKTIGGGD and Unacetylated Alpha tubulin peptide DGQMPSDKTIGGGD from SIGMA (USA) (resuspended in MilliQ water).

### Cell culture and treatments

Human embryonic kidney cells, HEK293T, human cervical carcinoma cells, HeLa, human breast cancer cells, MDA-MB 231, human lung adenocarcinoma cellsA549, chronic myeloid leukemia cells, K562, and human colon cancer cells, HCT11, were obtained from National Centre for Cell Sciences (NCCS), Pune, India. Cell line authentication by STR analysis was performed for 10 genetic loci for all the cell lines by Life code, Genomic technologies Pvt. Ltd., and tested for mycoplasma negative by performing PCR (mycoplasma specific genes) using EZdetect PCR kit for mycoplasma detection (CCK009). All the cells were cultured in high glucose DMEM medium containing 10% foetal bovine serum and 1% Penicillin/streptomycin (Himedia). Cells were seeded in 60 mm dishes and upon attaining 80–90% confluency, treatment was given with PCI-34051(Cayman Chemicals) (10 μM/20 μM) [12] and tubastatin (5 μM) for 24 h [12] or both in combination (PCI-34051 and Tubastatin) and Forskolin (Sigma) (10 μM) for 45 min independently along with untreated control as described earlier [13].

### Immunofluorescence

HeLa and HEK 293 T cells (Control, Forskolin, PCI-34051 (20 μM) treated) were fixed with 4% formaldehyde in PBS for 20 min at RT followed by permeabilization with 0.25% TritonX-100 in PBS. Nucleolus from HeLa cells isolated according to the standard protocol [14] were also fixed and permeabilized as above and immunofluorescence was performed as described previously [15].

### HDAC8 enzyme activity assay

HDAC8 enzyme activity assay was performed using HDAC8 FLUOR DE LYS fluorometric assay kit (Cat# BML-AK518–0001) for Control, PCI-34051(10 μM), Forskolin (10 μM), Tubastatin (5 μM) - treated and immunoprecipitated samples of either total, cytoplasmic and nuclear fractions of HeLa and HEK 293 T cells using anti-HDAC8 antibody and anti-HDAC6 (2 μg antibody for ~ 500 μg) according to the manufacturer’s protocol.

### Immunoprecipitation and MALDI TOF-TOF analysis

HeLa cells were trypsinized at 90% confluency, washed with 1XPBS and then lysed in IP Lysis Buffer (50 mM Tris pH 8.0, 150 mM NaCl, 10% Glycerol, 0.5% TritonX-100, 1X Protease Inhibitor Cocktail). Approx. 500 μg of total protein (in duplicates) was incubated with 2 μg of Anti-HDAC8 antibody and mouse IgG as an isotypic control at 4 °C for overnight. Protein A beads (20 μl) washed in lysis buffer (thrice) were added to the antibody-lysate mixture and further incubated for 2 h. After 2 h, beads were washed with MSWB Buffer (50 mM Tris pH 8.0, 150 mM NaCl, 1 mM EDTA and 0.1%NP-40) for three times at 4 °C. Equal (bead) volume of 2X sample buffer was then added and separated on 10% SDS PAGE along with the IgG input. The proteins on gel were visualized by Coomassie staining and differentially obtained bands in the IP sample was gel eluted and subjected to MALDI TOF-TOF analysis.

### Western blotting

Total, cytoplasmic and nuclear fractions of HeLa or HEK 293 T cells treated with or without PCI-34051 (20 μM), [12] tubastatin (5 μM) and/or forskolin (10 μM) were prepared as described previously [16]. Total protein samples of HEK 293 T, HeLa, MDAMB 231, K562, A549 and HCT11 cells / immunoprecipitated HDAC8 and HDAC6 samples from both HeLa and HEK 293 T lysates / HDAC8 siRNA transfected HEK 293 T and HeLa lysates were also prepared. The proteins were separated on 10 or 12% SDS-PAGE, transferred on to nitrocellulose membrane and probed with primary antibodies of HDAC8, Beta actin, Alpha tubulin, anti-acetylated alpha tubulin, HDAC6 and GAPDH. The bands were visualized using chemiluminiscence after probing with HRP-conjugated secondary antibody.

### Sequence analysis and homology modelling

The amino acid sequences of tubulins were retrieved from NCBI database (Accession numbers: AAH33064.1, AAH20946.1, and AAF34188.1 for alpha, beta and gamma tubulin respectively). They were further subjected to multiple sequence alignment using Clustal omega [17] to infer sequence conservation among the tubulin proteins. The sequences were analysed for functional conserved regions using Pfam database [18]. The crystal structures for beta-tubulin (PDB ID: 2XRP_A) and gamma-tubulin (PDB ID: 3CB2_A) were available in the database. However, Alpha-tubulin c did not have any structure in PDB and therefore its structure was built using homology modelling. Protein BLAST in eukaryotic genomes was carried out to analyse the sequence homology and identity. Homology modelling of Alpha tubulin was carried out by I-TASSER, an online webserver for protein structure prediction and structure-based function annotation [19]. The tertiary structure prediction was performed by I-TASSER server by using the best align template. Out of the generated models of the target sequence, the best template (PDB ID: 4I4T_A) was selected based on the significant sequence identity, C-Score, TM score and RMSD values of template structure.

### Protein-protein interaction studies

Protein-protein interaction studies of tubulins (alpha, beta, gamma) were carried out with HDAC8 (PDB ID: 1W22_A) using ZDOCK [20], which is an interactive online based server for docking protein-protein complexes.

### Purification of GST - hHDAC8

hHDAC8 (Full length) cDNA was amplified by using a polymerase chain reaction (PCR) with forward primer: 5′– GGAATTCATGGAGGAGCCGGAGGAACC-3′ and a reverse primer: 5’–CCGCTCGAGCTAGACCACATGCTTCAGATTCCC–3′. The PCR product was then sub cloned into pGEX- 6P1 plasmid. Clone was then overexpressed in *Escherichia coli* strain BL21 (DE3) with standardized expression conditions of 0.1 mM IPTG at 28 °C for 4 h. Induced culture pellet was lysed in 1XPBS buffer (140 mM NaCl, 2.7 mM KCl, 10 mM Na_2_HPO_4_, 1.8 mM KH_2_PO_4_) along with 1X Protease inhibitor cocktail, 1 mM PMSF, and 0.1 mM DTT. To the lysate 1% Triton X 100 is added and centrifuged for 20 min at a speed of 4000 rpm at 4 °C. Clear supernatant was used for binding with GST binding buffer (25 mM Tris pH 7.5, 300 mM NaCl, 1 mM EDTA, 1X Protease inhibitor cocktail) for 3 h on a rota spin at 4 °C. Binding was followed by collection of unbound and washes with 1X PBS (5 washes). Elutions were collected in 10 mM reduced glutathione, dissolved in 50 mM Tris pH 8.0. The purity of the protein was then analysed on SDS PAGE, followed by Western blot. Activity was then determined by HDAC activity assay.

### Fluorescence spectrometry studies

Binding studies of GST-HDAC8 & alpha tubulin peptides (ac & Unac) forms were carried out using Perkin Elmer precisely LS55 fluorescence spectrometry. Pure GST-HDAC8 (1-2 μM) was incubated with acetylated (500 nM) and unacetylated alpha tubulin peptides (500 nM) independently, with time dependence from 5 to 30 min, at 37 °C in Tris 50 mM pH 8.0. The decrease in the intrinsic fluorescence of GST-HDAC8 with both the conditions time dependently are read at 300–500 nm. The decrease in the fluorescence intensity was plotted against the wavelength (nm).

### Circular dichroism spectroscopy studies

Binding studies of GST-HDAC8 protein (2 μM) and acetylated (Lys40) or unacetylated alpha tubulin peptides (500 nM) were carried out on Jasco J-1500 (model L-1500-450) CD spectroscopy. Changes incurred in the secondary structure of GST-HDAC8 upon binding of the peptides were recorded as the mean ellipticity, with given set of parameters: start wavelength of 300 nm, end wavelength of 190 nm, scan speed of 100 nm/min, band width of 2 nm, cuvette cell size of 2 mm and at a temperature of 25 °C with two scan accumulations.

### In vitro deacetylation assay

In vitro deacetylation assay of custom synthesized acetylated (Lys40) alpha tubulin was carried out on HPLC (Schimadzu) with C18 (4.6 × 250 mm) column as described earlier [21]. Briefly, the unacetylated or acetylated tubulin peptides (500 nM) were incubated with purified GST-HDAC8 protein (2 μM) for 15 min at 37 °C in 50 mM Tris pH 7.5, 1 mM DTT buffer. The reaction was then quenched by addition of 1% TFA to the final reaction volume of 110 μl and injected into HPLC.

### RNA interference (siRNA) studies

Source for siRNA oligonucleotides were considered from [22] with sequence, HDAC8 Sense: GACGGAAAUUUGAGCGUAUUCUCU and Anti-sense: UAGAGAAUACGCUCAAAUUUCCGU. The oligonucleotides were converted into siRNA by following standard protocol as described earlier [23]. HeLa and HEK 293 T cells were transfected with HDAC8 siRNA (10–15 μg/100 mm dish) using lipofectamine 2000 (Invitrogen), and considering untransfected as control. Cells were harvested after 72 h of post-transfection and processed for total RNA and protein isolation.

### Real time analysis

HeLa and HEK 293 T cells treated with PCI-34051 (20 μM) or Paclitaxel (20 μM) for 24 h or HDAC8 siRNA (10–15 μg/100 mm dish) transfected HEK 293 T and HeLa for 72 h, along with control were subjected to total RNA isolation by using TRIZOL (Sigma-Aldrich, USA). As per the manufacturers protocol 1 μg of RNA was reverse transcribed with reverse transcription kit, (Invitrogen). Real-time RT-PCR was performed on Applied Biosystems StepOnePlus™ Instrument using KAPA SYBR® FAST qPCR master mix and gene-specific primers. The experiment was repeated twice, which were performed in duplicates. Fold expression determination, gene-to-GAPDH ratios were determined by using the 2^-∆∆Ct^ method. Details of the primers used are listed in the (Table. 1).

**Table 1:** List of Primers used for Real Time PCR Analysis

SNo	Gene	Sequence	Annealing Temp.(°C)	Amplicon size (bp)
1	GAPDH	FP: 5′ - GAGAAGGCTGGGGCTCATTTGC – 3’ RP: 5′ - TGGTGCAGGAGGCATTGCTGATG – 3′	61	145
2	HDAC8	FP: 5′ - GGCTAGGTTATGATGCCCAGC – 3’ RP: 5′ - CATGATGCCACCCTCCAGACC – 3′	60	141
3	HDAC6	FP: 5′ - TGTCTCTGGAGGGTGGCTACAAC – 3’ RP: 5′ - GGAAACTGAAGCCTGGGCACTC– 3′	60.5	125

### Cell migration assay

HeLa cells are grown in 6 well plates in duplicates till it attains 90% confluency and starved with serum free media for overnight. Using a sterile 200 μl tip wound is created by drawing straight lines at three different points in each well. Detached cells are aspirated and washed three times with 1XPBS. Cells are treated with PCI-34051 (10 μM & 20 μM) and Paclitaxel (10 μM & 20 μM) dissolved in 2% FBS respectively along with control (untreated). Migrated cells into the wound were examined by capturing images using Olympus CKX41, ProgRes CT3 phase contrast microscope after 24 and 48 h.

### Cell morphology analysis

HeLa and HEK 293 T cells are cultured in 6 well plates till attaining 70% confluency. Cells are starved for 12 h without serum and treated with PCI-34051 (20 μM) and Paclitaxel (20 μM) along with untreated control for 24 & 48 h. Examined difference in the morphology was captured by Olympus CKX41, ProgRes CT3 phase contrast microscope at 10X focussed lens.

### Cell cycle analysis

Cells (HeLa & HEK 293 T) were grown to attain 70% confluency and were serum starved for 12 h, followed by treatments with PCI-34051 (20 μM) and Paclitaxel (20 μM) respectively for 48 h and processed for cell cycle analysis.

### Statistical analysis

The statistical analysis was performed using one-way analysis of variance (ANNOVA) using GraphPad Prism software and statistical significance established (*p* < 0.05) was indicated by *.

## Results

### HDAC8 is localized predominantly to cytoplasm

To identify novel HDAC8 substrates, we have employed two different cell lines – cancerous cervical epithelial cells, HeLa cells, where HDAC8 is overexpressed and the other, non-cancerous HEK 293 T cells, considered as “normal” with basal level of HDAC8 expression.

**Fig. 1:**
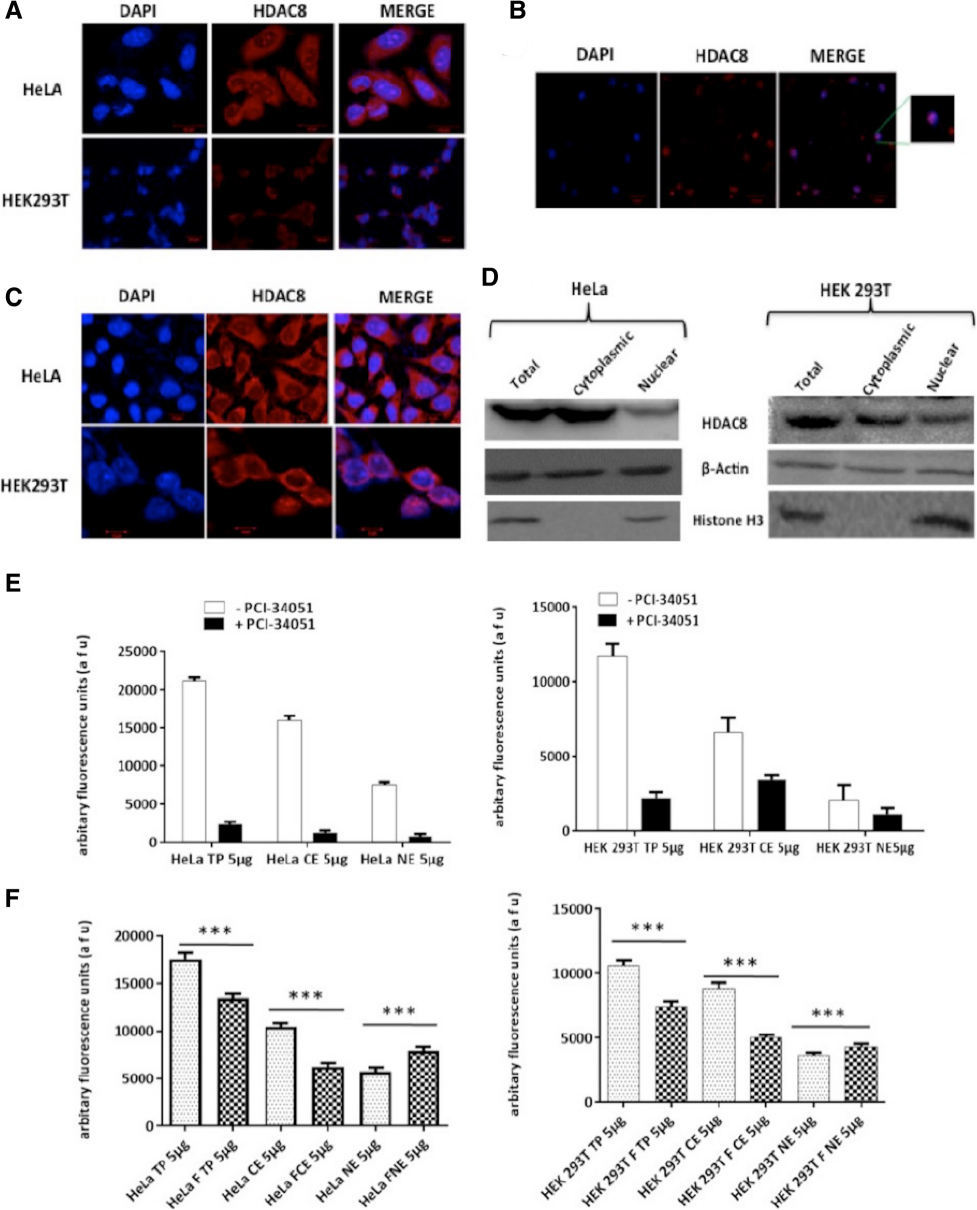
HDAC8 is predominantly cytoplasmic in localization. **a** Confocal images of HDAC8 localization in HeLa (upper panel) and HEK 293 T (lower panel) (Scale: 20 μm). **b** Nucleolar localization confirmation of HDAC8 in HeLa (Scale: 20 μm). **c** Confocal images of HDAC8 localization in Forskolin (10 μM) treated HeLa (upper panel) and HEK 293 T (lower panel) (Scale: 20 μm). **d** HDAC8 protein levels in total, cytoplasmic and nuclear fractions of HeLa and HEK 293 T. β-actin and histone-3 are loading controls. **e** HDAC8 enzyme activity assay with HeLa total (5 μg), cytoplasmic (5 μg) and (nuclear) 5 μg fractions in presence or absence of specific inhibitor PCI-34051(10 μM) in HeLa and HEK 293 T respectively. **f** HDAC8 enzyme activity assay with Forskolin (10 μM) treated, HDAC8 Immunoprecipitated fractions of total (5 μg), cytoplasmic (5 μg) and (nuclear) 5 μg fractions, along with untreated control fractions of HeLa and HEK 293 T respectively. * indicates *p*-value < 0.001

In the present study, the confocal microscopic analysis confirms the predominant cytoplasmic localization of HDAC8 in both HEK293T and HeLa cells (Fig. 1a) unlike other class I HDACs and our results are in agreement with the earlier studies [4, 24–26]. However, in HeLa cells, nucleolar expression of HDAC8 was also observed which was not seen in HEK293T cells (Fig. 1b). Such nucleolar localization of a protein involved in transcriptional regulation and gene repression in cancer cells was observed with CCCTC-binding factor (CTCF) [27]. Exploring the role of HDAC8 in nucleolus would be interesting and is not in the scope of this study.

HDAC8 is also known to be phosphorylated at Ser39 position by Protein kinase A (PKA) and its activity decreases with phosphorylation [13]. The class II HDACs are known to shuttle between cytoplasm and nucleus when phosphorylated. Therefore, we next analysed the cellular location of p-HDAC8 by treating cells with forskolin, an activator of PKA. The confocal images revealed no difference in localization of HDAC8 suggesting, HDAC8 and its phospho-form are majorly cytoplasmic in location (Fig. 1c).

The cytoplasmic localization of HDAC8 was further confirmed by immunoblot analysis using total, cytoplasmic and nuclear protein fractions of HEK 293 T and HeLa (Fig. 1d). HDAC8 enzyme activity assay using the immunoprecipitated fractions of total, cytoplasmic and nuclear proteins of untreated and PCI-34051- treated (a HDAC8 selective inhibitor [28]) HeLa [Fig. 1e (a)] and HEK 293 T [Fig. 1e (b)] cells demonstrated that both cytoplasmic and nuclear HDAC8 were active implicating its functional role both as histone and non-histone deacetylase. With forskolin treatment, there was significant increase in the nuclear fraction when compared to activity of untreated nuclear extract in both HeLa and HEK 293 T [Fig. 1f (a&b)].

### Alpha tubulin is a non-histone substrate of HDAC8

**Fig. 2:**
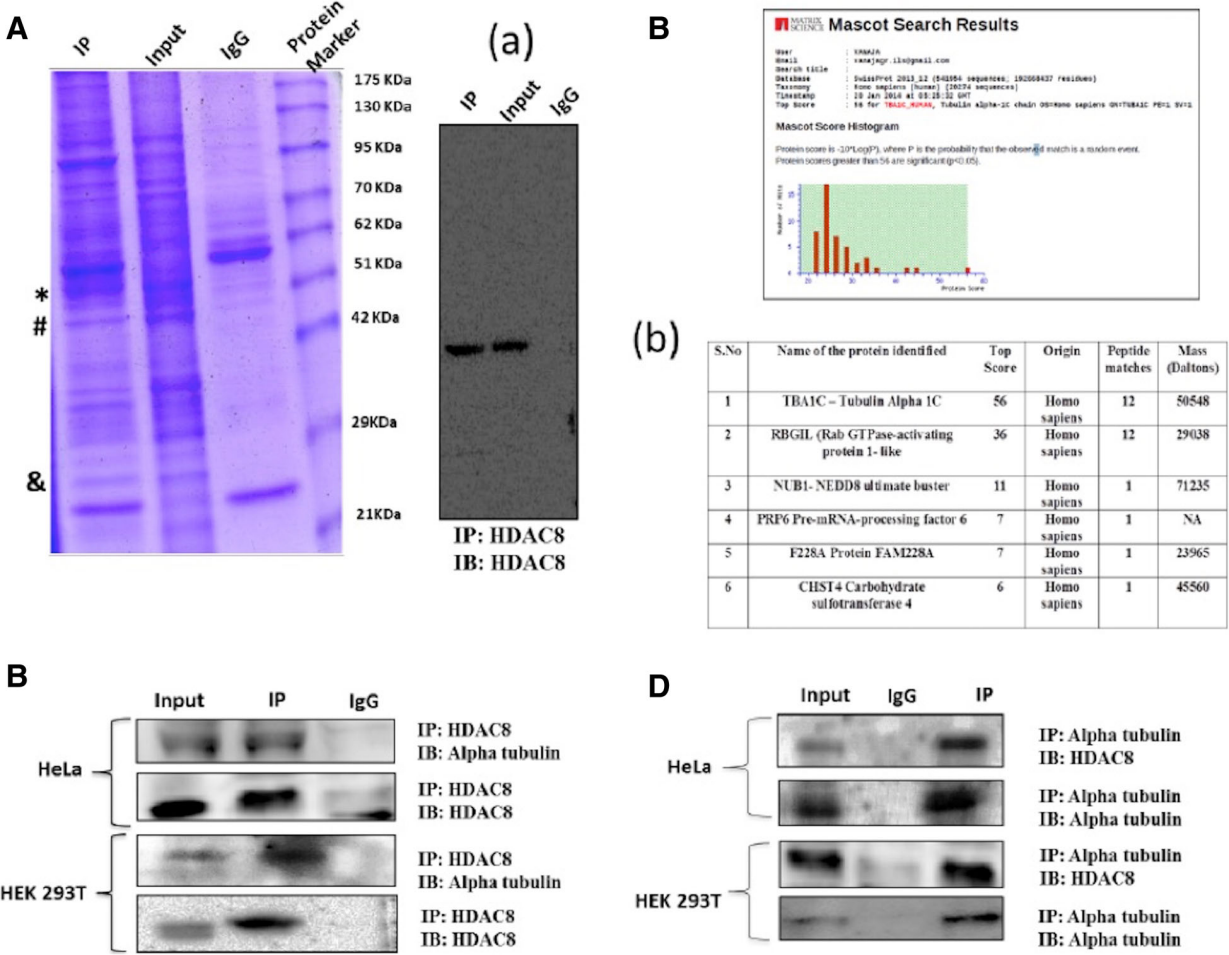
HDAC8 co-precipitates with Alpha tubulin. **a** Coommassie stained gel with input and HDAC8-immunoprecipitated HeLa cell lysate along with protein marker. Band at (*) ~ 50 kDa and at ~ 26 kDa (&) was excised and subjected to MALDI TOF-TOF analysis. (#) represents for HDAC8 in coomassie gel at 43 kDa, further confirmed by immunoblot. **b** MS analysis and Mascot search results confirmed TUBA1-C (Human Alpha Tubulin chain 1 C isoform) with highest topscore as the interacting partner of HDAC8. **c** Confirmation of HDAC8 and Alpha tubulin by immunoprecipitation using HDAC8 antibody in HeLa and HEK293T cells. **d** Reverse IP using Tubulin antibody confirms HDAC8 & Tubulin interaction in HeLa cells and HEK 293 T respectively

Identification of protein-protein interactions by mass spectroscopy followed by immunoprecipitation and co-IP experiments is a well-established and very useful technique [29]. Several interacting proteins have been identified using this approach. We therefore next tried to identify HDAC8 protein interacting partners by immunoprecipitation followed by MALDI-TOF-TOF analysis. Coomassie staining of the denaturing gel of immunoprecipitated protein samples from HeLa cells showed a very prominent band at approx 50 kDa (Fig. 2a). The protein was gel eluted and subjected to MALDI analysis. Mascot database search results identified protein as Alpha tubulin with a protein score greater than 56 and significant score of *p* < 0.05 (Fig. 2b). In view of cytoplasmic localization of HDAC8, we hypothesized Alpha-tubulin can indeed be a novel non-histone protein substrate of HDAC8. Co-IP (Fig. 2c) and reverse-immunoprecipitation (Fig. 2d) results confirmed that HDAC8 and Alpha tubulin do interact not only in cancer cells, HeLa but also in normal HEK 293 T cells.

### HDAC8 interacts only with alpha tubulin

**Fig. 3:**
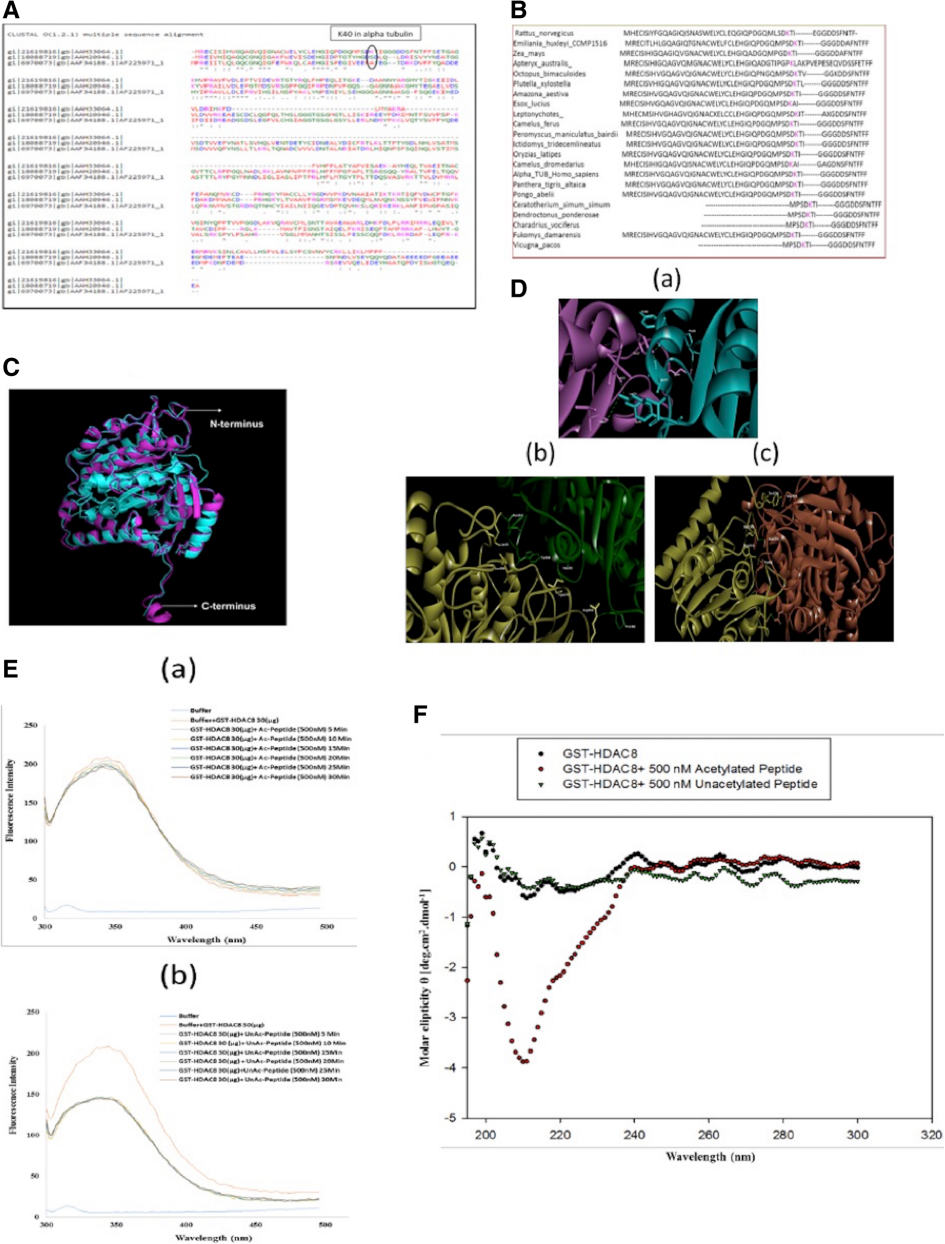
In silico analysis of tubulin isoforms and conserved Lys40 in alpha tubulin. **a** Clustal omega analysis showing Lys 40 of Alpha tubulin whereas, Beta and Gamma tubulins have serine and alanine respectively. **b** Lys40 position was found to be conserved in eukaryotes. **c** 3D crystal structures (generated by homology modelling) of Alpha, Beta and Gamma tubulins, used for studying protein protein interactions with HDAC8. **d** Protein protein docking studies of HDAC8 with Alpha, Beta and Gamma tubulins. **e** Fluorescence spectroscopy results of (a) GST-HDAC8 (30 μg) + ac-alpha tubulin peptide (500 nM) incubation from 5 to 30 min, decreases intrinsic fluorescence. (b) GST-HDAC8 (30 μg) + Unac-alpha tubulin peptide (500 nM) incubation from 5 to 30 min, no change in intrinsic fluorescence. **f** CD results for change in secondary structure for GST-HDAC8 alone, GST-HDAC8 (2 μM) + ac-alpha tubulin peptide (500 nM), and GST-HDAC8 (2 μM) + Unac-alpha tubulin peptide (500 nM), significant change in the secondary structure of GST-HDAC8 upon incubating with ac-alpha tubulin clearly signifies the more binding specificity for ac-alpha tubulin but not with unacetylated form

To understand the specificity of HDAC8 towards three isoforms of tubulin proteins, multiple sequence alignment of tubulin proteins using Clustal omega analysis was carried out (Fig. 3a). The analysis showed that Lysine residue at 40th position (K40) is only present in Alpha tubulin and that other tubulin forms lack this lysine residue. Further sequence analysis across different species in eukaryotes revealed the conservation of Lys40 in Alpha-tubulin protein indicating its functional significance (Fig. 3b). These results are in well-agreement with recently published study [30].

The functionally conserved K40 acetylation of Alpha tubulin and lysine deacetylase function of HDAC8, interested us to further evaluate the interaction of alpha tubulin and HDAC8. We used in silico approach to first confirm the protein-protein interaction. Since the crystal structure of Alpha tubulin of human was not available, a homology model was generated using I-D TASSER using 4I4T_A as the template structure. 4I4T_A is crystal structure of tubulin-RB3-TTL-Zampanolide complex from *Bos Taurus* [31]. It is a hexamer, therefore, Chain A was taken for modelling. The overall 3-D (three dimensional) structural arrangement of the crystal structure and model is conserved (Fig. 3c). More than 75% amino acid residues in model are in the allowed regions of the Ramachandran plot [32]. According to Verify-3D, the overall quality of the model was found to be 96.5% and ERRAT program shown that 71% residues had an averaged 3D-1D score > = 0.2. The RMSD between the template structure and model was 1.29 Å. C-score and TM-score were found to be 0.55 and 0.95 respectively within the acceptable ranges validating the model generated.

Protein-protein interaction studies of the tubulin proteins were carried out using HDAC8 (PDB ID: 1W22_A). The Alpha-tubulin model showed an interaction of Lys40 with Pro205 of HDAC8, including the IIe225, Val223, Tyr227 with Tyr225, Lys374, Asn372 and Tyr368 of alpha-tubulin and HDAC8 respectively. Out of Val223, Ile225 and Tyr227 residues of Alpha tubulin, it was observed that only Ile225 of Alpha tubulin is interacting with Tyr225 residue of HDAC8 (Catalytic region). As per interacting residues of HDAC8, Pro205 and Tyr225 residues belong to histone deacetylase catalytic domain [Fig. 3D (a)].

With respect to the beta-tubulin (PDB id: 2XRP_A), the residues Pro160, Val258, Trp344, Asn347 of beta-tubulin were found to be interacting with Arg356, Cys275, Leu308, Lys33 of HDAC8 respectively [Fig. 3D(b)]. Gamma-tubulin residues such as Tyr82, Ala370, Glu327, Asn79, Gln227 were found to be interacting with Pro273, Ser150, Tyr100, Cys352, Gln232 of HDAC8 [Fig. 3D(c)]. The ZDOCK score of the tubulins are provided in (Table. 2)**.** All the Figures related to protein-protein interaction studies were generated using Discovery Studio Visualizer version 4.1.

**Table 2:** ZDOCK Score for Alpha, Beta and Gamma tubulins

Protein Name	S_DS_ (k cal/mol)	S_ELEC_ (k cal/mol)	RMSD (Å)
Alpha Tubulin	−2.09	−2.73	1.45
Beta Tubulin	−3.14	−2.73	1.45
Gamma Tubulin	−2.87	0.10	1.55

*S*_*DS*_ indicates sum of Desolvation energy, *S*_*ELEC*_ indicates sum of Electrostatic energy, *RMSD* Root Mean Square Deviation of C-alpha atoms in Angstroms units

As the Desolvation and Electrostatic energy of Alpha tubulin is −2.09 and − 2.73 respectively (Range ≤ − 3 kcal/mol), followed by RMSD values which is 1.45 Å (Range ≤ 2.5 Å) for Alpha tubulin. This confirms a near native hit when compared to values of beta and gamma tubulin

Further in silico mutational studies were carried out to confirm the interaction between Lys 40 of Alpha tubulin and Pro205 of HDAC8. The Lys (40) of alpha tubulin was replaced with Arginine residue and Pro205 of HDAC8 with Alanine and protein-protein interaction with ZDOCK was performed. However, results did not demonstrate any potential interaction with these in silico amino acid replacements in both HDAC8 and Alpha tubulin due to which there was no figure generated in ZDOCK.

In vitro binding studies were performed for GST-HDAC8 (recombinant purified protein) and alpha tubulin peptides (ac, unac) using fluorescence and circular dichroism spectroscopy technique. Fluorescence spectroscopy analysis demonstrates the change in the intrinsic fluorescence of GST-HDAC8 when incubated with acetylated alpha tubulin time dependently compared to that of unacetylated alpha tubulin (Fig. 3E a&b). Circular dichroism data suggests that change in the secondary structure of GST-HDAC8 is due to binding of acetylated alpha tubulin, which was not observed with unacetylated tubulin (Fig. 3F).

### HDAC8 deacetylates alpha tubulin

**Fig. 4:**
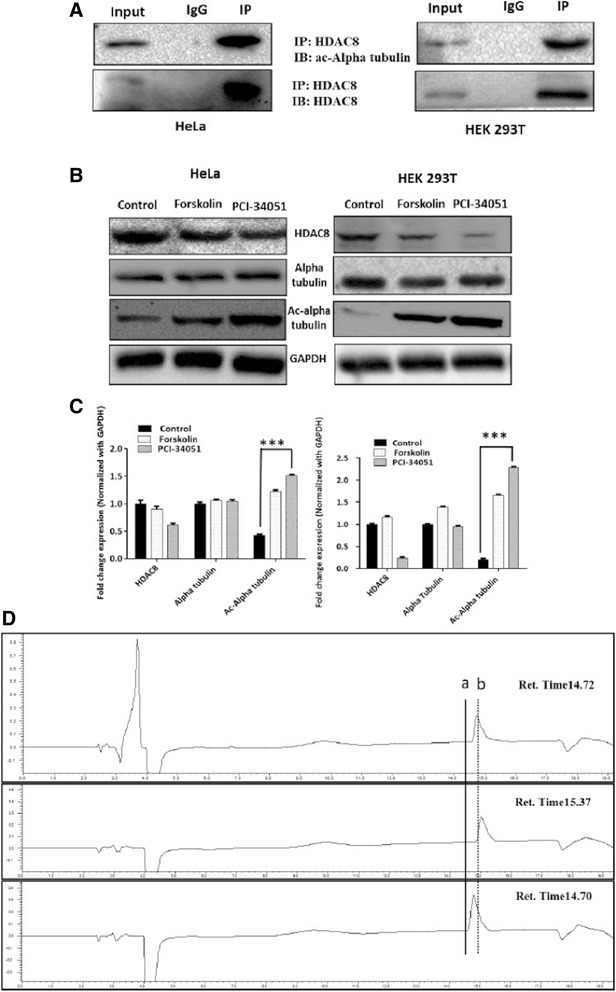
**a** HDAC8 interacts and deacetylates alpha tubulin. **a** HDAC8 and Acetylated Alpha tubulin Interaction in HeLa and HEK 293 T cells as demonstrated by immunoprecipitation using HDAC8 antibody followed by immunoblot analysis using ac-alpha tubulin. **b** ac-alpha tubulin levels increase when HDAC8 is inhibited with PCI-34051 (20 μM) or when treated with PKA activator, Forskolin (10 μM) and in HeLa and HEK 293 T cells. **c** Densitometry analysis of the immunoblot bands showing fold change in expression pattern of HDAC8, Alpha tubulin and acetylated tubulin normalized with GAPDH. **d** Lower panel of chromatogram represents for Unac-alpha tubulin peptide with a retention time of 14.70 Min. Middle panel of chromatogram for ac-alpha tubulin with retention time of 15.37 Min. Upper panel of chromatogram for GST-HDAC8 (30 μg) + ac-alpha tubulin (500 nM) with a retention time of 14.72 Min. Shift in the retention time from a (15.37) to b (14.72) clearly demonstrates the in vitro deacetylation of ac-alpha tubulin by GST-HDAC8

In silico and spectroscopy based results have confirmed the interaction of HDAC8 with alpha tubulin & acetylated alpha tubulin peptide. To further validate the functional interaction we carried out IP and co-IP experiments with HDAC8 and acetylated alpha tubulin (ac-tubulin) antibodies. The immunoblots clearly showed the HDAC8 and ac-tubulin interaction (Fig. 4a). The levels of ac-tubulin increased significantly when cells were treated with forskolin (Fig. 4b) (known to inactivate HDAC8 by phosphorylation) indicating HDAC8 as a tubulin deacetylase. Densitometry results for the fold expression change of acetylated alpha tubulin normalized with GAPDH (Fig. 4c), clearly demonstrates ac-alpha tubulin as one of the HDAC8 substrate.

To further confirm the deacetylation of ac-tubulin by HDAC8, we carried out HPLC-based deacetylation assay and the HPLC results clearly demonstrated the in vitro deacetylation of acetylated alpha tubulin (Lys40) peptide by GST-HDAC8 with a significant peak shift in the retention time corresponding to unacetylated peptide (Fig. 4d).

### HDAC8 might be primary tubulin deacetylase in HeLa cells

**Fig. 5:**
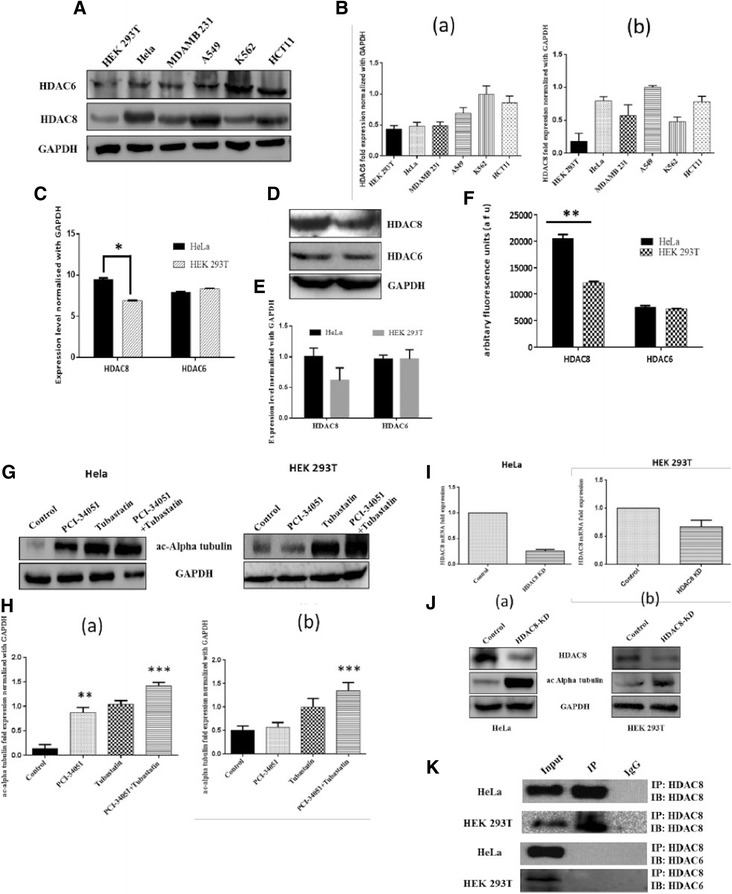
HDAC8 might be primary tubulin deacetylase in HeLa cells. **a** Immunoblot showing the protein expression levels of HDAC8 and HDAC6 in various cancer cell lines. **b** Densitometric analysis of the (a) HDAC6 bands and (b) HDAC8 bands of immuoblot A. **c** Real time analysis confirms higher HDAC8 expression compared to HDAC6 in HeLa and HEK 293T normalized with GAPDH * indicates p-value <0.0001. **d** Protein expression pattern of HDAC8 & HDAC6 in HeLa and HEK 293T. **e** Densitometric analysis of bands in Immunoblot D. **f** HDAC enzyme activity assay performed with IP Anti-HDAC8 & IP Anti-HDAC6 with HeLa &HEK 293T lysates respectively. * indicates *p*-value <0.0001. **g** Increased ac-alpha tubulin levels in presence of PCI-34051 (20 μM) when compared to tubastatin μM treated condition in HeLa, whereas, tubastatin (5 μM) treatment in HEK 293T increases ac-alpha tubulin levels compared to PCI-34051 (20 μM) treated condition. **h** Densitometry graphs of the immunobands in G. **i** Real time PCR analysis of the mRNA expression of HDAC8 in HEK 293T and HeLa upon siRNA transfection. **j** Immunoblot analysis of HDAC8 and ac-Alpha tubulin in HDAC8 knockdown HeLa (a) and HEK 293T (b) cells. **k** IP-Co-IP studies of HDAC8 & HDAC6 in HeLa and HEK 293T

HDAC6 is a tubulin deacetylase [33]. However, our experimental results also suggested HDAC8 as tubulin deacetylase. In order to understand these results, we have evaluated the expression of HDAC6 and HDAC8 in HEK 293 T, HeLa, MDA-MB-231, A549, K562, and HCT11 cell lines. Results demonstrated that there was no significant difference in HDAC6 expression, when compared between HEK 293 T, HeLa and HCT11. Whereas, A549, K562 and MDA-MB 231 cells have shown two fold higher expression when compared to control HEK 293 T (Fig. 5A and B (a)). On the other hand, HDAC8 is over expressed in HeLa, A549 and HCT11 compared to HEK 293 T (Fig. 5A and B (b)). Based upon the expression pattern of HDAC6 in different cancer cell lines, we presume its role in deacetylating ac-alpha tubulin in HeLa might be taken over by HDAC8 due to its significant overexpression compared to HDAC6.

Next, we have evaluated mRNA and protein levels and HDAC activity of HDAC6 and HDAC8 in HeLa and HEK 293 T cells. The results clearly demonstrated the dominance of HDAC8 in HeLa cells when compared to normal HEK 293 T. On the other hand, there was no significant difference in the HDAC6 expression levels in HeLa and HEK 293 T cells (Fig. 5C, D, E & F). These results indicate that HDAC6 might be primary tubulin deacetylase; however, the increased HDAC8 in HeLa cells might be taking over the function of HDAC6 as tubulin deacetylase. However, further experiments in various cells showing differential expression of HDAC6 and HDAC8 are warrented.

Next, we tried to determine the effect of HDAC8 and HDAC6 expression on acetylation of tubulin in HEK293T and HeLa cells using specific inhibitors, PCI-34051 and Tubastatin alone or in combination (PCI-34051 + Tubastatin). The increased acetylated alpha tubulin levels in HeLa under PCI-34051 treated conditions when compared to control, clearly demonstrates the possible role of HDAC8 as tubulin deacetylase in HeLa (Fig. 5G and H). The combination treatment (upon inhibition of both HDAC6 & HDAC8) has shown a clear impact on acetylation of alpha tubulin, which can be explained due to the synergistic effect of dual inhibition of HDAC6 & 8. In HEK 293 T, there was no significant increase in PCI-34051 treated condition, whereas HDAC6 inhibition resulted in increase in ac-alpha tubulin levels implicating HDAC6 as main tubulin deacetylase in HEK 293 T cells  (Fig. 5G and H (b)).

Further we examined the effect of HDAC8 knockdown on acetylation pattern of alpha tubulin using siRNA in both HeLa and HEK 293 T. Real time PCR data confirmed the decreased mRNA expression of HDAC8 in both the cell lines upon siRNA transfection (Fig. 5I). Immunoblot results clearly demonstrated the impact on acetylation pattern of alpha tubulin in both the cell lines [Fig. 5J (a&b)]. This significant increment in the acetylation of alpha tubulin in HDAC8 KD (siRNA knockdown) samples when compared to control, justifies the role of HDAC8 as one of the tubulin deacetylase in HeLa cells. Finally, IP and reverse-IP results of HDAC8 and HDAC6 in HeLa and HEK 293 T further demonstrate their independent mode of activity (Fig. 5K).

### HDAC8 alters alpha tubulin functionality

**Fig. 6:**
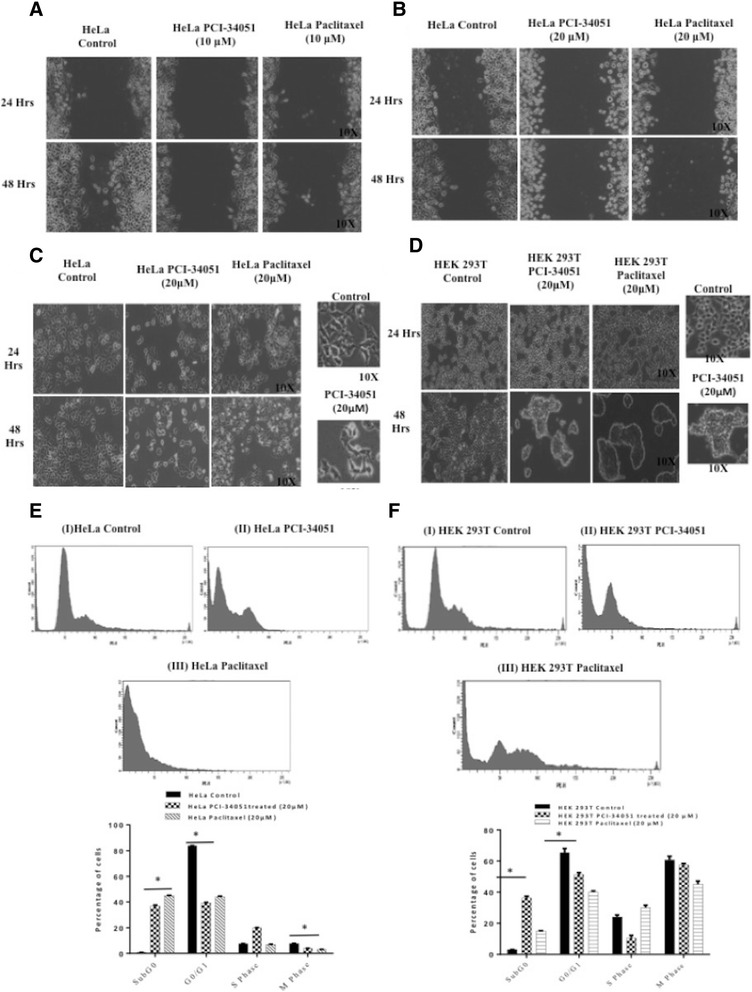
HDAC8 alters Alpha tubulin Functionality. **a** Cell Migration Assay in HeLa cells in absence or presence of PCI-34051 (10 μM) or Paclitaxel (10 μM) Migration efficiency: Control> > Paclitaxel>PCI-34051. **b** Cell Migration Assay in HeLa cells in absence or presence of PCI-34051 (20 μM) or Paclitaxel (20 μM). **c** Cell morphology analysis demonstrating reduced (shrinking) cell size of HeLa with PCI-34051 (20 μM) or Paclitaxel (20 μM) at 24 & 48 h. **d** Cell morphology analysis in HEK 293 T cells representing clump formation with PCI-34051 (20 μM) or Paclitaxel (20 μM) at 24 & 48 h signifying role of HDAC8 in microtubule organization. **e** Cell cycle analysis of HeLa, showing (I) control, (II) PCI-34051 (20 μM) and (III) Paclitaxel (20 μM) treated condition. **f** Graphical representation of subG0, G0/G1, S and M phase in HeLa, deciphering significant effect of PCI-34051(20 μM) on Mitotic phase. * indicates *p*-value < 0.05

The results from cell migration assay clearly demonstrated that migration efficiency was significantly decreased in HeLa cells when HDAC8 is inhibited with PCI-34051 upto 48 h with a notable decrease in cell size (Fig. 6a & b).

To further validate the difference in the cell morphology, HeLa and HEK 293 T cells were treated with PCI-34051 and Paclitaxel, tubulin polymerization inhibitor, a positive control, for 24 and 48 h. A clear distinguished difference in the cell morphology, with decreased cell size (shrinking) in HeLa, and cell aggregation in HEK 293 T was observed in both treated conditions when compared with control (Fig. 6c & d).

Cell cycle analysis of HeLa cells treated with PCI-34051for 48 h showed an increased sub G0/G1 peak along with a significant (*p* < 0.05) reduction in mitotic phase of cell cycle when compared to untreated control (Fig. 6e). On the other hand, HEK 293 T normal cells did not show any mitotic phase reduction when HDAC8 was inhibited indicating that HDAC6 might be the primary tubulin deacetylase. However, a significant increase in the sub G0/G1 phase was observed in HEK293T cells treated with PCI-34051 which can be attributed to the inhibition of other normal physiological functions of HDAC8 (Fig. 6f).

## Discussion

HDAC8 belongs to class I HDACs that are generally localized to nucleus and regulate gene expression. Although HDAC8 is well-characterized structurally, its precise cellular loaclization, functional role and the substrates are not known completely [1]. Since HDAC8 is known to be involved in female-specific cancers such as ovary, cervical and breast [10], we have used HeLa cervical cancer cells along with HEK 293 T normal cells so as to get an insight on its role in normal and cancer cells. HDAC8 is known to deacetylate acetylated peptide in vitro implicating its unique behaviour when compared to other class I HDACs that function in protein complexes. In order to better understand this unique nature and identify the protein interacting partners of HDAC8, it is therefore important to first determine the precise cellular localization of HDAC8. Results from our studies clearly demonstrate major cytoplasmic localization of HDAC8, attributing its functional role in deacetylating both histone and non-histone proteins. Further, the cytoplasmic HDAC8 activity decreased when treated with forskolin, but nuclear HDAC8 enzyme activity increased with forskolin indicating that phospho-HDAC8 might have a role in nucleus such as protecting human ortholog of the yeast ever-shorter telomeres 1B (hEST1B) from ubiquitination [34]. Evidence from our immunoprecipitation and MALDI TOF TOF, followed by immunoblotting studies further confirms Alpha tubulin as a non-histone interacting partner of HDAC8 irrespective of cancerous (HeLa) and normal cells (HEK 293 T).

In silico characterization studies further demonstrated, the uniqueness of conserved K40 position in alpha tubulin which is known to be acetylated in normal cellular process and is involved in regulation of protein trafficking, cell cycle, and cell migration [30]. Protein-protein interaction studies, with all the three forms of tubulins further validated the importance of alpha tubulin with higher number of interactions, especially Lys40 (a potential acetylation site) of alpha tubulin (catalytic region) with that of Pro205 of HDAC8 (catalytic region). Importance of Lys40 position for its potential interaction with HDAC8 was further validated by in silico mutational analysis (Lys40 to Arg in alpha tubulin & Pro205 to Ala in HDAC8) which revealed loss of interaction between two proteins. Fluorescence spectrometry is widely used in studying the molecular (binding information) and structural changes in the protein, which is affected by either protein-protein interaction or protein with small molecule interactions. The significant change which occurs around the microenvironment of the aromatic amino acids like Trp, Tyr and Phe are known to induce changes in the intrinsic fluorescence emission properties of the protein, which are measured and analysed [35]. HDAC8 has Trp-4, Phe-13, and Tyr-21 residues respectively. Significant decrease in the fluorescence quenching from 5 min to 30 min, refers the prominent protein peptide binding interactions. Unacetylated peptide did not show any notable changes in the fluorescence intensities. Circular dichroism studies revealed a significant change in the secondary structure, when incubated with acetylated alpha tubulin, without any notable differences with that of unacetylated form.

IP and Co-IP results for acetylated alpha tubulin with HDAC8, along with functional regulation studies of acetylation levels of alpha tubulin, under forskolin treated condition (phospho-form of HDAC8, known to be less active compared to control) reveals significant increase of acetylated tubulin expression, validating it as a novel substrate for HDAC8, in addition to HDAC6, which is an established tubulin and known to be a target in many of the cancers [36, 37]. Considering the positive results from our binding studies of GST-HDC8 - ac, unac peptides, in vitro deacetylation assay carried out with GST-HDAC8 incubation with acetylated alpha tubulin peptide (Lys40), clearly admits for its substrate.

As our results clearly demonstrate the role of HDAC8 in deacetylating acetylated alpha tubulin, question arises why this functional redundancy between HDAC6 and HDAC8. So, we first analysed the expression of HDAC6 and HDAC8 in normal and cancerous cervical tissue samples in The Human Protein Atlas database and learned that HDAC6 expression is low in cervical cancer tissues when compared to normal cervix (medium expression) and that HDAC8 is overexpressed (low-medium expression) in cancerous cervix when compared to undetectable expression in normal cervix [38]. We have confirmed the expression of HDAC6 in different cancer cell lines of various origins i.e., HeLa, HCT11, A549, K562 & MDAMB 231. There was no significant change in the expression of HDAC6 in between HEK 293 T, HeLa and HCT11 cell lines, when compared to A549, K562 & MDA-MB 231. Whereas, HDAC8 expression was observed to be high in HeLa, A549, HCT11 & MDA-MB 231. These immunoblot results are in agreement with human protein atlas data, suggesting differential expression pattern of HDAC6 & HDAC8 in different cancer cell lines, assigning their specific/independent roles in deacetylating their target substrates. Further, results from our studies state dominant expression of HDAC8 in HeLa cells to that of HEK 293 T, when compared to HDAC6 expression, which did not vary significantly in both cell lines further confirming with the human protein atlas database. These results increased the probability of considering HDAC8 in taking over the function of a tubulin deacetylase in HeLa, compared to HDAC6. To further confirm this, inhibition studies were carried out. HDAC8 inhibition (PCI-34051) contrastingly increased the acetylation of alpha tubulin in HeLa, when compared to HEK 293 T. Dual inhibition of both HDAC6&8 further increased the acetylation in a synergistic manner attributing to their independent role in deacetylating acetylated alpha tubulin. HDAC8 knockdown studies, confirms the importance of HDAC8 role as one of the tubulin deacetylase in HeLa cells.

Tubulins are one of the major cytoskeletal proteins and consists of alpha, beta and gamma tubulin proteins in the family [39]. Acetylated alpha tubulin is known to stabilize microtubules and effect intracellular transport, cell migration [40] and primary determinant for cell morphology [41]. HDAC8 inhibition impedes the migration property in HeLa in turn affecting the morphology. Shrinkage of cell size in HeLa and clump formation in HEK 293 T may be due to disruption in the cytoskeletal machinery in maintaining cell structure. The effect was quite comparable to that of paclitaxel treated condition which was considered as a positive control for tubulin inhibition. This can be explained due to the functional role of HDAC8 in deacetylating the acetylated alpha tubulin in HeLa, which is considered as a mark for stabilization of microtubules. Degree of tubulin acetylation is also known to affect the cell motility by altering the microtubule dynamics [42]. Owing to the important role of microtubules in cell cycle, inhibition of tubulin polymerization with inhibitors such as paclitaxel or inhibition of tubulin deacetylation results in cell-cycle arrest and cell death [43]. Our results demonstrate the significant (*p* < 0.05) reduction in mitotic phase in HeLa treated with PCI-34051, when compared to HEK 293 T that justifies the role of HDAC8 in HeLa as predominant deacetylase of alpha tubulin, which is in turn involved in regulating cell cycle in G2/M phase. Whereas, no major significant change in the mitotic phase of HEK 293 T condition can be explained due to the availability of HDAC6 that might be functioning as tubulin deacetylase even upon HDAC8 inhibition.

## Conclusions

**Fig. 7:**
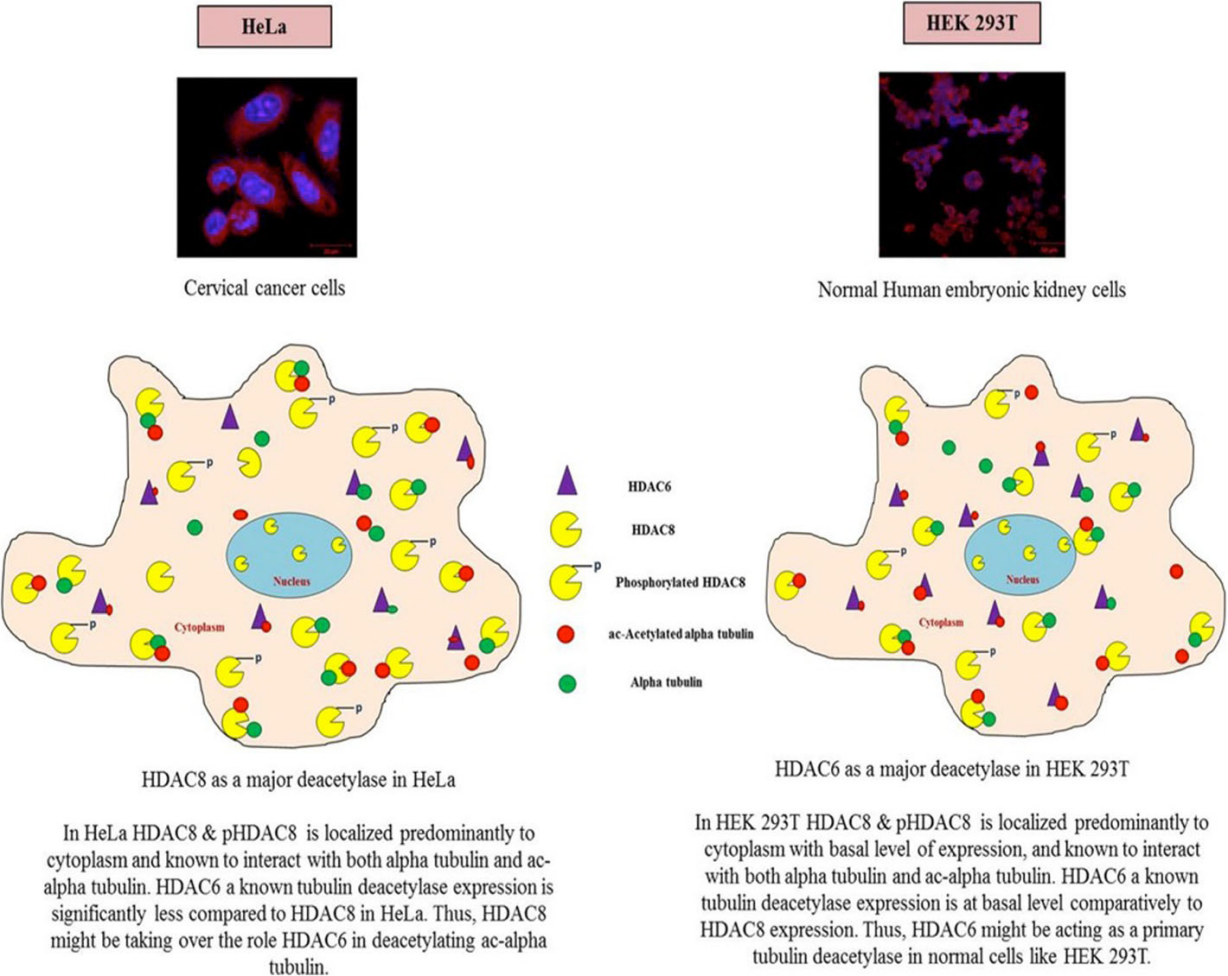
Schematic representation of overall summary

Overall, the study objective was to identify novel HDAC8 substrates and the results of our study clearly signify HDAC8 as a tubulin deacetylase. Although HDAC6 is a proven tubulin deacetylase and HDAC6 inhibition results in cancer cell death, its expression is not altered in cervical cancer as evident by the mRNA, protein expression and enzyme activity studies. Therefore, inhibition of HDAC6 in these tumours might result in undesired side effects. The study demonstrated that inhibition or knockdown of HDAC8 in HeLa cells, leads to hyper acetylation of tubulin which in turn stabilizes the microtubules and inhibit cell migration and mitotic phase of cell cycle. Our study signifies the role of HDAC8 as tubulin deacetylase in cervical cancer cells and therefore might be a better target in these cancers (Fig. 7).
